# Moribund Patients in Hospital

**Published:** 1908-10-03

**Authors:** 


### Moribund Patients in Hospital.

Hospitals are no longer a horror, but there remain many small details in their administration and in their policy which are open to criticism. Some of these points are matters on which only the expert can rightly judge. But there are others on which everyone, whether layman or expert, is entitled to be heard, and in the discussion of which the common feelings of humanity—of which a hospital is, after all, only the practical expression—must be allowed to overweigh other, and it may be less sentimental, considerations. One of these questions is the treatment of moribund patients in our charitable institutions.

No one who has had any connection with hospital life can for a moment credit the assertion, still made by the wilfully ignorant, that daily attendance upon the sick and suffering blunts the finer susceptibilities of those whose duty it is to see most of what is terrible and gloomy. Rather will he agree that such constant attendance whets the deeper sympathy with suffering, and refines the feeling which Keats, who gauged so truly and described so finely "the weariness and the fret" of hospital life, styled the most ennobling of desires—the wish to lighten and assuage, tempered by the knowledge that no aid or help beyond sympathy can avail. For all that, there are phases of hospital life which may strike those who are acquainted with them and accept them as matters of routine far less forcibly than the man who comes to know them for the first time. The screens drawn round the bed of the dying ward-patient, the relatives and the priest passing into the shadow of the curtains—all this on the one side: on the other, the sister cutting up the midday joint and the patients playing dominoes on the balcony. The picture is not over-drawn and the contrast is not made intentionally ludicrous. We have all seen them at one time or other, felt a passing twinge, and wondered, for a moment at least, that such a state of things is still allowed to exist. That no change has been made so far is due largely to the fact that no one has drawn attention to the matter, and that the wonder of a moment has not crystallised into action. At most of our institutions the dying patient is treated exactly as he was treated a hundred years ago. The limited privacy of a few screens is considered sufficient to veil him from his ward fellows, as if hospitals had to act up to Marshal Ney's remark that a man can die anyhow and anywhere. In one, at least, of our large London hospitals, the attendants still carry into the ward a special bier for the removal of the body, cover it with a special pall, and march down the stairs with it and across the courtyard where the convalescent patients are. In the interests of the dying patient such little attentions as we plead for are probably superfluous. He, at least, is in unison with Ney. But in the interests of his friends and relatives, and, above all, in the interests of his fellow patients, it is highly desirable that death should be isolated from a hospital ward as much as possible.

In Continental hospitals this desirability has been recognised and is daily becoming more appreciated. There the modern institution has no hospital bier, no death-bed screen—nothing, in fact, that can attract the attention of the other patients to what is going on at one particular point in the ward. The body is removed on an operating-room wheel-couch, covered with a sterilised towel, just as if the patient was being taken to the theatre. It is not carried down the ordinary stairs, but reaches the mortuary through an underground passage. Even more effective is the system of the "Dying Room" now being generally adopted in the newer institutions. Attached to two or more wards, but entirely isolated from them, is a special room reserved for such cases as are moribund. The patients are removed from the general ward, without attracting attention, and placed in this extra room: here they can be attended by their relatives, and here, too, they can be prepared for final removal to the mortuary. By means of this system, which entails comparatively little extra trouble, the patients in a general ward are spared the distressing experience of witnessing a series of preparations which to many may appear ill-omened, and to all painful. We sincerely trust that some such method may ere long be tried in our own institutions.

